# A dual-rule encoding DNA storage system using chaotic mapping to control GC content

**DOI:** 10.1093/bioinformatics/btae113

**Published:** 2024-02-28

**Authors:** Xuncai Zhang, Baonan Qi, Ying Niu

**Affiliations:** College of Electrical Information Engineering, Zhengzhou University of Light Industry, Zhengzhou 450000, Henan, China; College of Electrical Information Engineering, Zhengzhou University of Light Industry, Zhengzhou 450000, Henan, China; College of Building Environment Engineering, Zhengzhou University of Light Industry, Zhengzhou 450000, Henan, China

## Abstract

**Motivation:**

DNA as a novel storage medium is considered an effective solution to the world’s growing demand for information due to its high density and long-lasting reliability. However, early coding schemes ignored the biologically constrained nature of DNA sequences in pursuit of high density, leading to DNA synthesis and sequencing difficulties. This article proposes a novel DNA storage coding scheme. The system encodes half of the binary data using each of the two GC-content complementary encoding rules to obtain a DNA sequence.

**Results:**

After simulating the encoding of representative document and image file formats, a DNA sequence strictly conforming to biological constraints was obtained, reaching a coding potential of 1.66 bit/nt. In the decoding process, a mechanism to prevent error propagation was introduced. The simulation results demonstrate that by adding Reed-Solomon code, 90% of the data can still be recovered after introducing a 2% error, proving that the proposed DNA storage scheme has high robustness and reliability.

**Availability and implementation**: The source code for the codec scheme of this paper is available at https://github.com/Mooreniah/DNA-dual-rule-rotary-encoding-storage-system-DRRC.

## 1 Introduction

With the rapid development of the Internet and the Internet of Things, a vast amount of digital data is continuously generated and accumulated. It is estimated that by 2025, nearly 175 zettabytes (ZB) of data will be generated. Modern storage systems face challenges such as high energy consumption, elevated costs, and inadequate data security. DNA, an ancient and efficient information carrier within living organisms, has garnered the attention of researchers due to its attributes of long-term stability, low energy consumption, high security, and immense storage potential. Compared to conventional information carriers, DNA molecules offer numerous advantages, including exceptionally high storage density. Research has demonstrated that the data storage capacity of 1 g of DNA can reach up to 215 petabytes (PB), ∼22 544 384 000 gigabytes (GB), equivalent to the data storage of 220 000 1TB hard drives (De Silva and Ganegoda 2016). Its distinctive stability enables DNA molecules to be preserved for thousands of years under suitable conditions, such as temperatures below −20°C, humidity ranging from 20% to 40%, protection from light, and vacuum conditions (Allentoft *et al.* 2012, Bhat 2018).

So far, numerous methods utilizing organic molecules (DNA, RNA, oligopeptides, metabolites) for information storage have been proposed (Anavy *et al.* 2019, Cafferty *et al.* 2019, Choi *et al.* 2019, Kennedy *et al.* 2019, Koch *et al.* 2020). The advancement of DNA sequencing technology over the past few decades has made genome sequencing increasingly accessible and widespread. As a result, DNA information storage has emerged as a highly esteemed storage strategy. The traditional DNA storage process typically entails the conversion of binary information from files into DNA base sequences. Subsequently, these encoded sequences are synthesized, assembled, and stored within appropriate mediums to ensure both stability and accessibility. Retrieval and interpretation of the original file information are achieved through the utilization of sequencing technologies.

Although the early codebook ([00, 01, 10, 11] → [A, T, C, G]) significantly increased the encoding capacity of DNA, it introduced specific structural patterns within the nucleotide sequence, leading to particular challenges during synthesis and sequencing processes (Kosuri and Church 2014, Yazdi *et al.* 2015, Shendure *et al.* 2017). For instance, excessively long repeated nucleotide sequences (*n* > 4 nt) can lead to base errors during DNA sequence synthesis or sequencing. Uneven distribution of GC content in DNA sequences, whether too low or too high, can result in uneven distribution during polymerase chain reaction (PCR) amplification, and there is a preference for GC content during sequencing, i.e. regions on the genome with a GC content of around 50% are more likely to be sequenced (Niedringhaus *et al.* 2011, Ross *et al.* 2013, Van der Verren *et al.* 2020). The presence of palindrome subsequences within DNA sequences can lead to secondary structures like hairpins during PCR amplification, ultimately affecting the efficiency of the amplification process. Therefore, the DNA code should adhere to specific rules to reduce systematic errors during the DNA data storage process. In August 2012, Church *et al.* from Harvard University (Church *et al.* 2012) achieved DNA storage of HTML files, JPG images, and JavaScript programs with 5.2 MB of data. They first proposed a “bit-base” mapping codebook, where each bit corresponds to one base, which avoids homopolymers of length > 3. However, the data could not be completely recovered due to the limited sequencing depth and the lack of error correction strategies. In contrast, Goldman *et al.* (2013) added a compression strategy and parity-check codes to increase robustness to errors in the DNA storage system. In 2015, Grass *et al.* (2015) proposed a new method for coding and decoding “symbol-codon” inner and outer codes. These strategies come at the expense of storage density. Erlich and Zielinski (2017) proposed a novel DNA storage method based on Fountain code (LT code) in 2017 and achieved 2.15 MB data files (including Text, OS, Image, PDF, Movie, and Software) for DNA storage and entirely accurate recovery, which does not require repeated sequencing and significantly reduces redundancy compared to previous coded decoding methods. DNA Fountain introduces screening constraints of low redundancy, homopolymer length, and GC content to improve the information fidelity and achieves a storage density of 1.57 bit/nt using the Luby transform. However, there is a risk of decoding failures when dealing with a particular binary due to the fundamental problem of the Luby transform (Lu *et al.* 2009). Ping *et al.* (2022) proposed a yin-yang coding and decoding system (YYC) to encode two binary bits into one base using both yin and yang coding rules. This coding method is robust and highly compatible with synthesis and sequencing techniques, but it has to be filtered from multiple coding schemes. Welzel *et al.* (2023) proposed a coding scheme that supports variable-sized coding sequences to correct substitutions, insertions, losses, and whole DNA strand errors. DNA-Aeon was reported to have better error correction at similar levels of redundancy and reduced DNA synthesis costs compared to other DNA storage technologies.

Nowadays, there are still some difficulties in the development of coding algorithms, i.e. ensuring coding efficiency while needing to take into account the satisfaction of specific constraints and establishing personalized coding for arbitrary constraints. In this article, we propose a DNA dual rule rotational coding (DRRC), where the two coding rules complement each other, the GC content can be stabilized at 50% by the control of chaotic sequences, and the introduction of rotational coding mode effectively avoids the emergence of long homopolymers and has a low time complexity. Through simulation, it is proven that the scheme presented in this article can store source files of different formats and types with a logical storage density of 1.66 bit/nt and has high robustness to ensure data security.

This article is organized as follows. Section 2 describes the constraints of DNA-based storage coding. Encoding and decoding methods are proposed in Section 3. Simulation results are given in Section 4, and Section 5 concludes the article.

## 2 Constraints in DNA storage

### 2.1 GC content constraints

DNA storage requires a stable and homogeneous DNA molecule, which consists of four bases, A, T, C, and G. Guanine and cytosine pair with each other in the form of hydrogen bonds to form a stable pairing relationship, and this base pairing is the basis for the transmission of genetic information and DNA replication. GC content refers to the ratio of the sum of the lengths of guanine and cytosine to the total length of the sequence of DNA. The content constraints must satisfy the range of the chemical assay, typically 40% < GC < 60%. For a sequence S, GC(S) can be expressed as shown in Equation (1), where $\left|G+C\right|$ is the sum of the number of G and C in the sequence and $\left|S\right|$ is the total length of the sequence (Cao *et al.* 2022).

(1) GCS=G + CS

Ross *et al.* (2013) conducted a systematic comparison of the GC bias of four sequencing technologies: Complete Genomics DNA nanoball sequencing, Illumina sequencing by synthesis, Ion Torrent semiconductor sequencing, and Pacific Biosciences. The results consistently showed that good coverage was obtained at the mid-range of the GC content of the sequences and showed a decrease in coverage for both high and low GC content sequences. Ion Torrent semiconductor sequencing showed that up to 30% of genomes with low GC content were not covered at all (Quail *et al.* 2012), while library preparation failed completely for genomes with high GC content (Bragg *et al.* 2013). Although Illumina HiSeq platform was slightly superior, it exhibited a coverage bias for sequences with extreme GC content that could not be combined with data from different platforms to correct.

### 2.2 Run length constraints

The length formed by the recurrence of each base in a sequence is called the run length, and during the synthesis and sequencing of DNA molecules, repeated bases may lead to experimental failure. Particularly in sequencing, as the homopolymer length increases, the insertion error rate and deletion error rate increase for all platforms except PacBio RS where the insertion error rate is consistently high, and the most sensitive platform to homopolymer is the Ion Torrent PGM, which was found to produce no reads for homopolymers longer than 14 nucleotides in length in one study (Quail *et al.* 2012). The run length constraint is defined as:

(2) Bi≠Bi+1, i∈1, n-1

where $B_{i}$ belongs to a base in a DNA sequence $\left(B_{1},\;{\;B}_{2},\;{\;B}_{3}\ldots B_{n}\right)$ of length *n*. When the maximum run length is >6, the substitution and deletion error rate increases (Schwartz *et al.* 2012). This also leads to high error rates in the DNA storage process.

## 3 DNA dual rule rotary encoding storage system

In nature, the DNA molecule consists of two complementary polymer chains, and the complementary nature of the DNA double-stranded structure enables it to form a stable double-helix structure, which can ensure the accuracy and stability of the genetic information in the transmission process. Inspired by the double-stranded structure, this article proposes a new DNA storage system that uses chaotic mapping to control the GC content of base sequences and builds a dual-rule coding table based on the rotational coding of Goldman *et al.* (2013) to conform to the constraints in the storage of DNA, map binary sequences to base sequences. This storage program can effectively balance the GC content and avoid the generation of homopolymers, enhance the stability of the DNA sequence, and greatly reduce the error rate during sequencing.

### 3.1 Principles and features of DRRC

**Figure 1.**
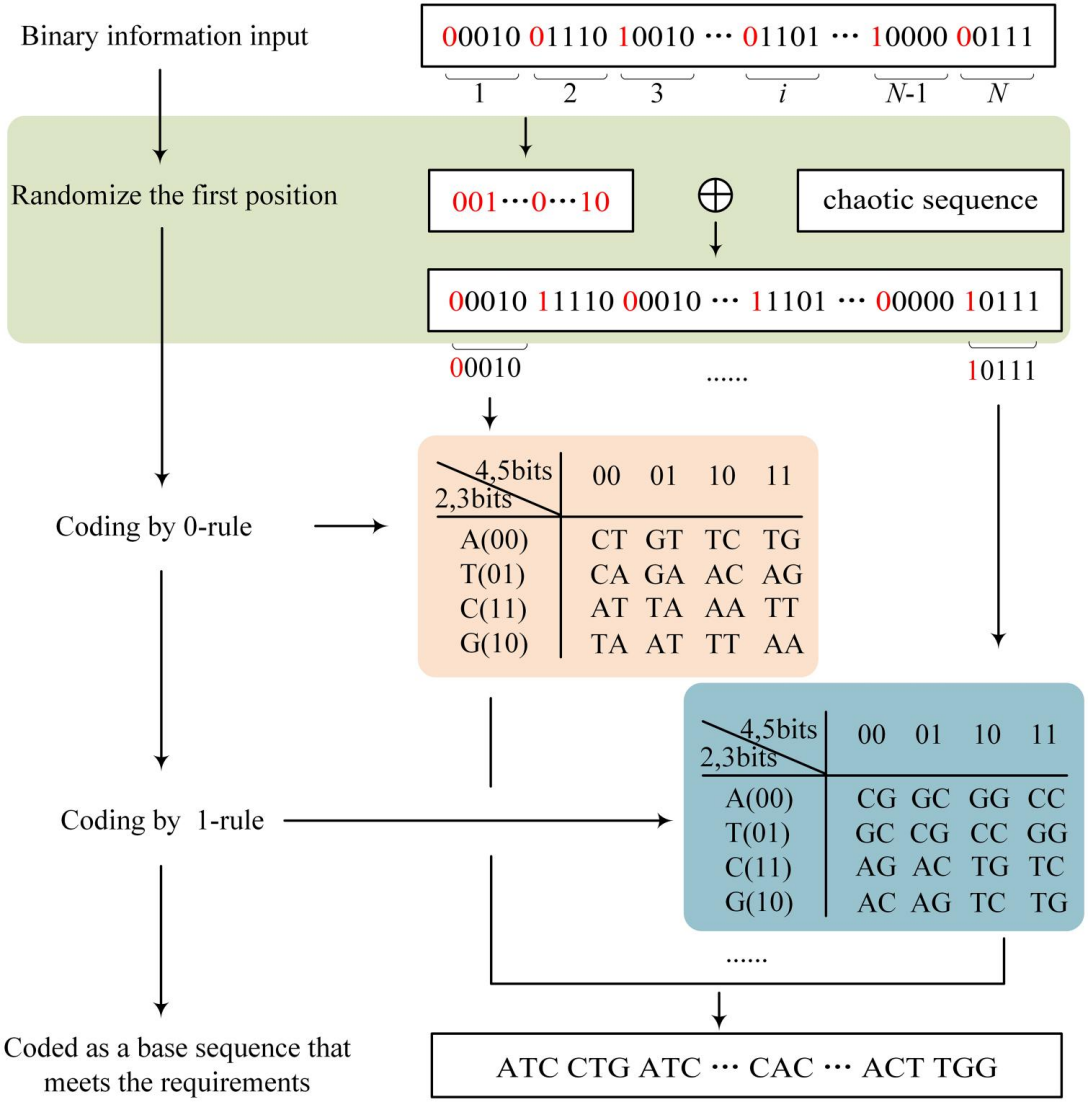
DRRC coding process.

As shown in Fig. 1, the principle of DRRC is to divide the binary stream into groups of five bits each, and the first bit of each group is used to control the selection of coding rules, if the first bit has a value of 0, the remaining four bits are coded with the 0-rule, and if the first bit has a value of 1, the remaining four bits are coded with the 1-rule. The coding rules are shown in Fig. 1. The 0-rule coding table constructed in this article has a GC content of 1/3 for all codewords, and the 1-rule coding table has a GC content of 2/3 for all codewords. To balance the GC content of base sequences obtained from the final coding, the value of the first bit of each group of the original binary stream must be controlled. Therefore, before encoding, the first bit of every five bits is extracted to form a sequence. To ensure an equal count of 0 and 1 in this sequence, it is iteratively exclusive OR with a chaotic sequence until 0 and 1 are balanced. The balanced sequence is replaced back to its original position. The altered binary stream is encoded with double rules. Due to this unique encoding method of DRRC, we can control the GC content of the base sequence flexibly. The first bit of every five bits in the binary is not involved in the encoding, the storage density is significantly increased.

DRRC consists of four steps:

Step 1: The binary data is grouped every five bits, and the first bit of each group is randomized to obtain a binary sequence with the first bit 0/1 balanced.

Step 2: Deriving a dual-rule coding table based on the established constraints and coding mapping the binary sequence derived in step 1.

Step 3: DNA synthesis and storage.

Step 4: DNA sequencing and decoding for error correction.

### 3.2 Randomization of the first position

Chaotic sequences are acyclic, i.e. there are no repeating patterns in the sequence. Although chaotic sequences exhibit complex and irregular behavior, the values of the sequences are uniquely determined for a given mapping or equation and initial conditions. In this article, a one-dimensional logistic mapping is used as in Equation (3), μ is the control parameter, which is set to a fixed value of 3.9 in this scheme. $X_{n}$ is in the range of (0, 1), and the chaotic sequences obtained from the one-dimensional logic mapping need to be binarized by Equation (4), and converted to the corresponding binary sequences to facilitate the subsequent first position randomization. To get the correct chaotic sequence when decoding, three parameters of the one-dimensional logical mapping need to be stored, which are the control parameter 3.9; the length of the chaotic sequence; and the initial value at the time of successful iteration. Encoding these three parameters according to Blawat’s scheme yields a DNA sequence of about 115 nt, and 200 nt when error-correcting and indexing bits are taken into account (Blawat *et al.* 2016).

(3) Xn+1=μ×Xn×1-Xn

(4) Xi'=1, if Xi≥0.50, if Xi≤0.5

**Figure 2.**
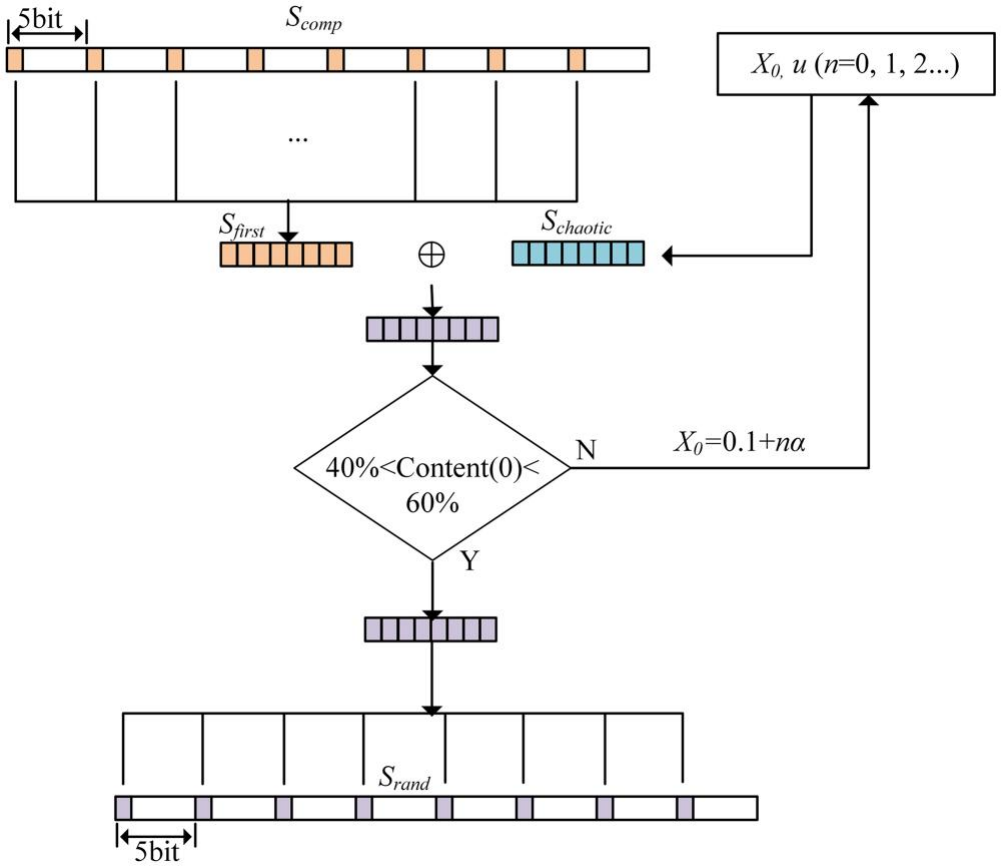
Randomization of the first position.

The binary sequence to be encoded is *S_comp_*. It needs to control the ratio of 0 and 1 at the first position to balance the GC content of the subsequent encoding so that it falls within 0.5 ± α, where α is controlled by *Content0*. Figure 2 shows the process of randomizing the first position. Firstly, *S_comp_* is grouped every five bits, and the first bit of each group is taken out and arranged to form a binary sequence called *S_first_*. Then, a one-dimensional logistic map is used to generate a chaotic sequence that is the same length as *S_first_*, called *S_chaotic_*. *S_first_* and *S_chaotic_* are iteratively exclusive OR until a sequence with a 40%–60% content of 0 or 1 is obtained. Finally, this sequence is replaced with the first position of the original sequence, resulting in the randomized binary sequence *S_rand_*. Figure 6D demonstrates that the number and time of iterations in the first position randomization always remain within acceptable limits.

The randomization process is not only effective in controlling the GC equilibrium but also improves the security of the original message. Chaotic systems are highly sensitive and unpredictable, and their outputs exhibit some degree of irregularity and complexity similar to random sequences. The random sequence generated by the chaotic system is used as a key to encrypt the plaintext, thus achieving the purpose of encryption (Materassi and Basso 2008).

### 3.3 Dual-rule DNA coding

This section is about mapping binary sequences to base sequences, grouping binary sequences in 5-bit units, and mapping them to base sequences via a table of dual-rule codes to satisfy the constraints of a maximum run length of 3 and GC balancing.

Assuming that the binary sequence after randomization of the first position is *S_rand_*, for each group of mapping process $\left\{S_{i1},{\;S}_{i2},\;S_{i3},\;S_{i4},\;S_{i5}\right\}\to \left\{N_{i1},\;N_{i2},\;N_{i3}\right\}\;(1<i<n,\;N_{i1},\;N_{i2},\;N_{i3}\in \{A,\;T,\;C,\;G\})$, Equation (6) determines the mapping rules for $S_{i2},\;S_{i3},{\;S}_{i4},{\;S}_{i5}$ based on $S_{i1}$.

(5) Srand=S11, S12,  S13, S14, S15,…Sn1, Sn2, Sn3, Sn4, Sn5

(6) Si1, Si2, Si3, Si4, Si5→0-ruleNi1, Ni2, Ni3, if Si1=0Si1,  Si2, Si3, Si4, Si5→1-ruleNi1, Ni2, Ni3, if Si1=1

The above equation shows that $S_{i1}$ is the benchmark for selecting the bi-mapping rule, after the first position randomization, the proportion of 0 in the sequence ($S_{11},{\;S}_{21},{\ldots \;S}_{i1},\ldots {\;S}_{(n1)1},{\;S}_{n1}$) is $\frac{\sum_{i}^{n}S_{i}}{n}\approx 50\%$. To ensure that the mapped bases are GC balanced, under the 0-rule, it is necessary that only one of the mapped bases $\left\{N_{i1},{\;N}_{i2},{\;N}_{i3}\right\}$ is either G or C base. Under the 1-rule, it is required that only two of the mapped bases $\left\{N_{i1},{\;N}_{i2},{\;N}_{i3}\right\}$ are either G or C. Based on the above, the dual-rule coding table is constructed as shown in Fig. 1.

(7) Ni1=A, if Si2Si3=00T, if Si2Si3=01C, if Si2Si3=11G, if Si2Si3=10

The first bit is not involved in the mapping under the 0-rule and 1-rule coding. Bits 2 and 3 are mapped to position 1 of the base sequence, as shown in Equation (7).

Rotary coding (Goldman *et al.* 2013) is highly respected as one of the most important techniques widely used in data communication and storage. The method considers GC content and homopolymer length constraints, which largely reduces the errors occurring during data storage. Thus, with the idea of rotational coding, bits 4 and 5 of the binary are mapped to positions 2 and 3 of the base sequence, and according to Fig. 1, the result of its mapping is determined by the value of itself and the previous base together.

Take the binary sequence “01101001001001001110” for example, it is first grouped in units of 5 bits, then the first bit is extracted to form the sequence “0010,” and iteratively exclusive OR with the chaotic sequence generated by the logic mapping, and the 0/1 content of the result of the exclusive OR is calculated until the 0/1 content of the sequence reaches the range of 49%–51%, then the iteration is stopped. After calculating the chaotic sequence after one iteration satisfies the above constraints, at which point the chaotic sequence is “1011” (*µ, x*_0_). The result of the exclusive OR is “1001,” replaced by a bit back to the original position. At this point, the sequence to be encoded is “11101001000001011110.” $S_{11}=1,S_{21}=0,S_{31}=0,S_{41}=1$, Then the remaining positions within each chunk are mapped according to the 1-, 0-, 0-, and 1-rules, respectively. The mapping result “CACTCAATCCTG” is obtained, in which the maximum homopolymer length is <3, and the GC content is 50%.

### 3.4 Decoding algorithms

The decoding algorithm is the inverse of the encoding algorithm, and the key to decoding is the process of segmenting the base sequence (in slices of 3) and counting the GC content within each slice.

Suppose the received base sequence is *N_receive_* = (*N*_11_, *N*_12_, *N*_13_, … *N_n_*_1_, *N_n_*_2_, *N_n_*_3_), *N_i_*_1_, *N_i_*_2_, *N_i_*_3_ ∈ {A, T, C, G}. Firstly, count the sum of the number of G/C $n_{i\mathrm{GC}}$ in the *i*th slice $N_{i1},{\;N}_{i2},{\;N}_{i3}$, The value of $n_{i\mathrm{GC}}$ is used to determine which rule is used to decode the *i*th slice. As shown in Equation (8), when the number of GCs in the *i*th slice $n_{i\mathrm{GC}}=1$, it is decoded with the 0-rule, and when the number of GCs in the ith slice $n_{\mathrm{iGC}}=2$, it is decoded with the 1-rule.

(8) Ni1, Ni2, Ni3→0-ruleSi1, Si2, Si3, Si4, Si5, if niGC=1Ni1, Ni2, Ni3→1-ruleSi1, Si2, Si3, Si4, Si5, if niGC=2

Another key to decoding is replacing the bit of the first position; after decoding by the 0, 1 inverse rule, the binary sequence is still not the original sequence, first split the binary sequence (in units of 5) and extract the first bit, exclusive OR with the chaotic sequence saved in the first position randomization process (*μ*, *x*_0_) to get the original first position sequence, and then replace the original first position sequence back to the first bit of each slice to get the correct original binary sequence.

The whole decoding process can be divided into the following two steps:

Step 1: Segment the base sequences obtained from sequencing according to the fixed bit number 3, and select the decoding rules for decoding according to the value of $n_{i\mathrm{GC}}$.

Step 2: Split the binary sequence obtained by step 1 according to the fixed number of bits 5, extract the first bit to form the sequence ${(S}_{11},{\;S}_{21},\ldots \;S_{n1})$, exclusive OR with the chaotic sequence to get $S_{\text{first}}$, and replace each bit of it with $S_{i1}$ in order.

## 4 DNA sequence design and simulation analysis

**Figure 3.**
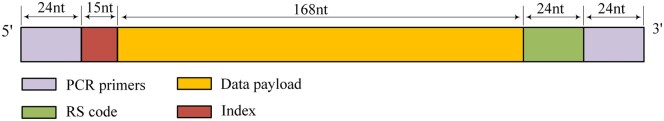
Oligonucleotide sequence design for DRRC.

Figure 3 shows the structure of the DNA sequence designed in this scheme, the payload portion is 168 nt, and the left and right sides with a length of 24 nt are primers, which play a role in amplifying the target DNA fragments in the PCR, and also affect the quality of the sequencing. The Reed–Solomon (RS) code is a linear packet code that corrects coding and decoding random errors during the process and is widely used in DNA storage (Wicker and Bhargava 1999). Therefore, this scheme introduces the RS code, which is the green part of the figure with a length of 24 nt. The red part with a length of 15 nt is the index, which is used to identify the DNA sequence position.

To better evaluate DRRC storage solutions. In this article, DRRC is compared with Church *e al.* (2012), Goldman *et al.* (2013), Grass *et al.* (2015), DNA Fountain (Erlich and Zielinski 2019), and YYC (Ping *et al.* 2022) using Chamaeleo (Ping *et al.* 2021), a platform developed by the Beijing Genomics institution Research Institute for pooling different DNA storage methods. DNA Fountain and YYC algorithms used their original settings with constraints of 40%–60% GC content and maximum homopolymer length 4. Several coding and decoding tests were performed afterward, and detailed analyses of the DNA sequence characteristics of the different schemes mentioned above, as well as the robustness of the error propagation of the DRRC, were performed.

### 4.1 DNA sequence characterization

**Figure 4.**
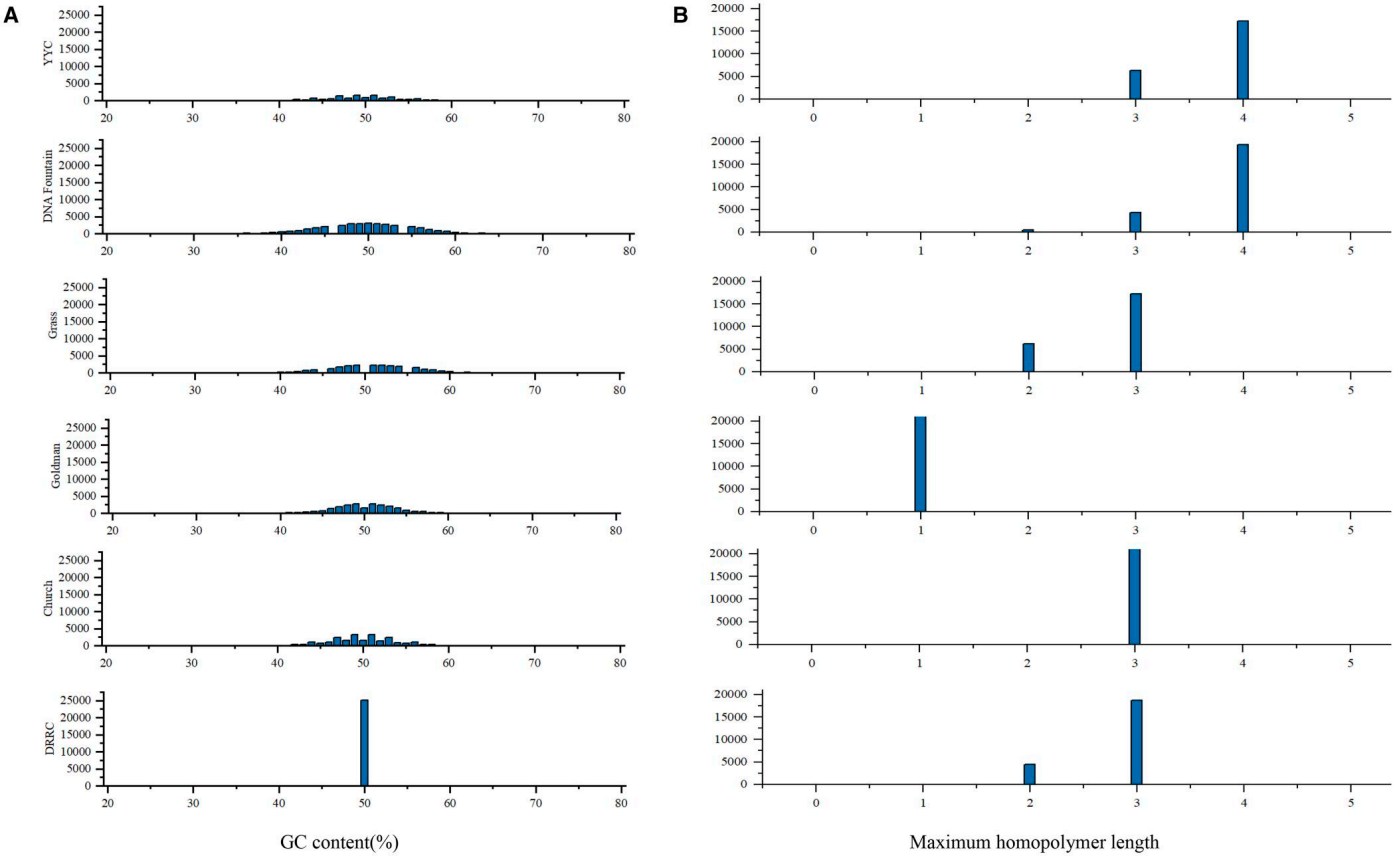
Comparison of the properties of DNA sequences by Church, Goldman, Grass, DNA Fountain code, YYC, and DRRC. (**A**) Distribution of GC content. (**B**) Distribution of maximum homopolymers.

In this section, DRRC is combined with five other classical coding schemes, such as Church, Goldman, Grass, DNA Fountain, and YYC, to encode the PDF file of the English version of *Wandering Earth* and *Pride and Prejudice*, with a total of 876 960 bytes of data. In the encoding process, the binary sequence obtained from reading is divided into segments every 280 bits, totaling 25 056 segments. Each binary sequence was encoded independently in each encoding scheme to obtain 25 056 base sequences, and finally, the sequence characteristics of all base sequences were counted. Figure 4A shows the distribution of GC content under different coding schemes. This scheme can control the GC content of DNA sequences at 50% by controlling the 0/1 proportion of the first position sequence (*Content0* = 50%), YYC and DNA Fountain always control the GC content at 40%–60% due to their unique screening mechanism, Church’s scheme has a GC content of 34%–66%, Goldman’s 21%–80%, and Grass’s 31%–70%. As shown in Fig. 4B, in terms of homopolymerization, the scheme of this article is comparable to Grass, Church, with most of the maximum homopolymer lengths clustered at 3 nt, and the remainder at 2 nt. DNA Fountain and YYC have a maximum homopolymer length of 3–4 nt. It is worth noting that Goldman’s maximum homopolymer length is 1 nt, in this scheme, if the current base is the same as the previous base, the current base is replaced to achieve the effect of avoiding homopolymerization, but this model increases redundancy at the expense of net information density. In summary, this solution provides coding patterns that are reliable and stable, which facilitates the subsequent DNA synthesis, PCR, and sequencing processes. This feature not only reduces the occurrence of accidental errors to a certain extent but also improves the efficiency of the data reading process (i.e. PCR and sequencing).

### 4.2 Analysis of error correction capacity

The DNA storage process inevitably introduces errors that pose a serious challenge to the accurate recovery of DNA data. DNA molecules may be subject to base substitution, insertion, and deletion errors during synthesis; at the PCR amplification stage, substitution errors may occur; the probability of substitution error for a single base on the 454 GS Junior sequencing platform is 0.05%, and the probability of insertion/deletion is 0.39%, the probability of substitution error for a single base on the second-generation sequencing platform Illumina MiSeq is 0.24%, and the probability of insertion/deletion is 0.009%; the probability of substitution error for a single base on the third-generation sequencing platform Pacific Biosciences RS is 1.10286%, and the probability of insertion/deletion is 15.56571% (Quail *et al.* 2012, Bragg *et al.* 2013, Ross *et al.* 2013). Therefore, the inclusion of error correction measures in decoding is critical to the integrity of data recovery.

(9) Rrecovery= NsdseqNtseq

**Figure 5.**
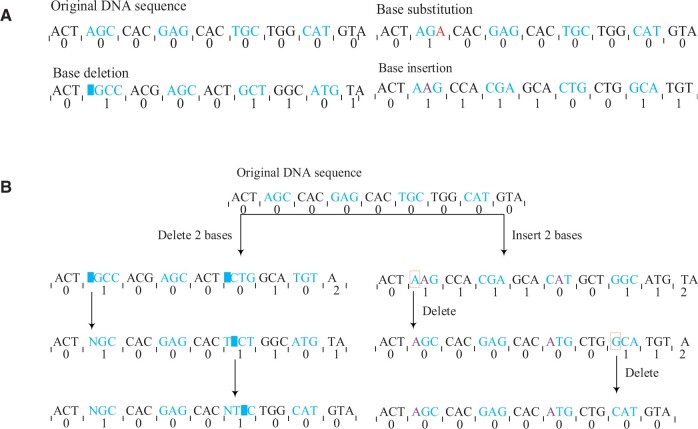
Blocking error propagation in DNA sequences. (**A**) Effect of single-base errors on the Hamming distance of slices. (**B**) Mechanisms for detecting errors and blocking error propagation using the Hamming distance.

**Figure 6.**
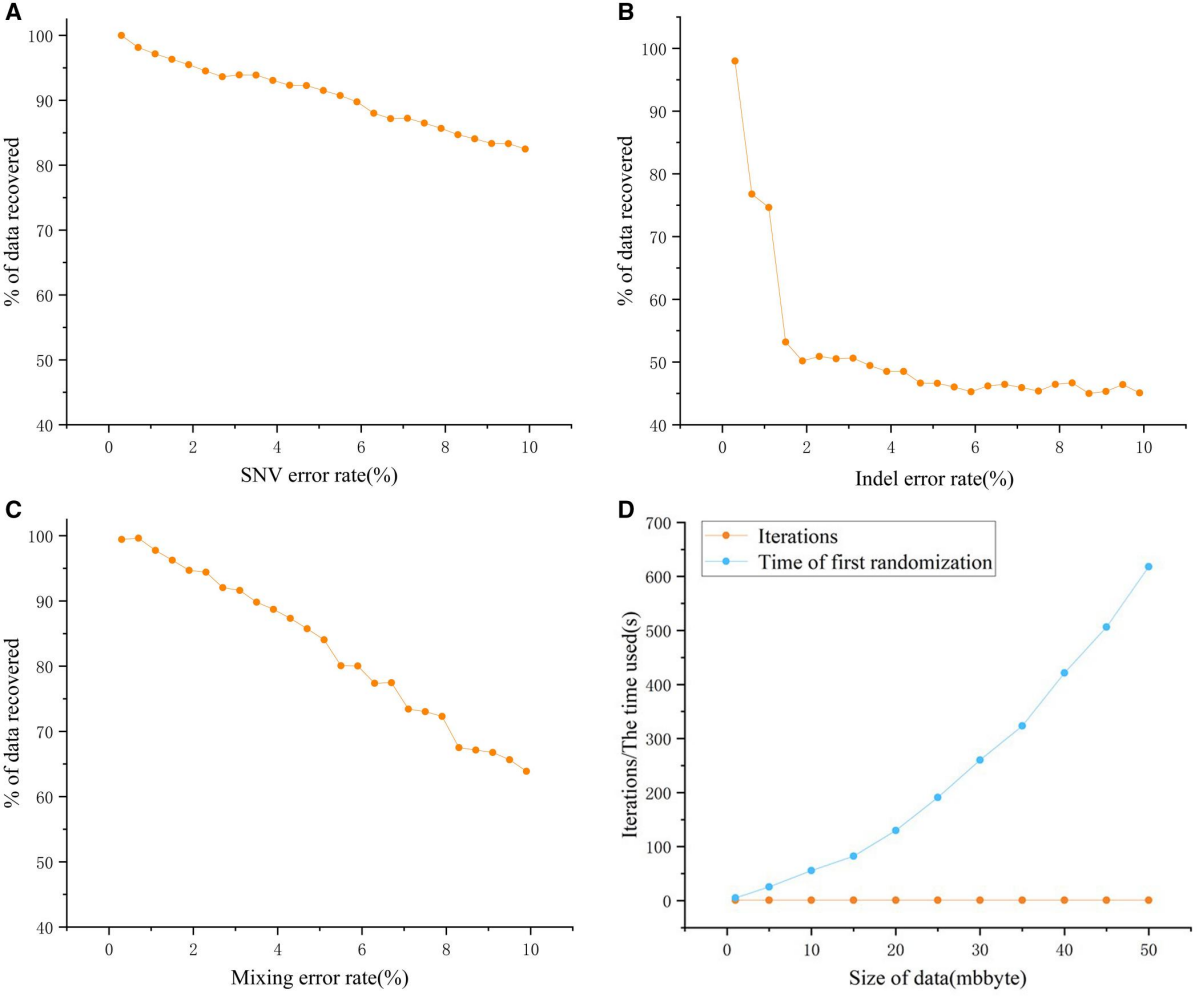
Error correction capability and coding efficiency analysis. (**A**) Variation of data recovery rate with the introduction of substitution error rate without adding any error correction measures. (**B**) Variation of data recovery rate with the introduction of insertion/deletion error rate without any error correction measures. (**C**) Variation of the data recovery rate with the introduction of mixing error rate after the inclusion of the blocking error propagation mechanism and RS code. (**D**) Time variation of randomization of the first position sequence from 1 to 50 MB.

The data recovery rate is calculated as shown in Equation (9). $N_{\mathrm{sdseq}}$ is the number of fully recovered sequences and $N_{\mathrm{tseq}}$ is the overall number of DNA sequences. Without incorporating any error correction measures, a *Mona Lisa* image of size 97.53 kb was taken as input and encoded with a dual rule to form 2316 base sequences of length 207 nt. Substitution and insertion/deletion errors of 0.1%–10% were randomly introduced in them. As shown in Fig. 6A and B, the data recovery rate is always above 80% with the introduction of 0.1%–10% substitution errors, and decreases rapidly and remains at 50% with the introduction of 0.1%–10% insertion/deletion errors. This is because the decoding of DRRC is done in slices (three bases to a slice), as shown in Fig. 5A, and substitution errors will only affect the decoding of this slice itself and will not affect the subsequent slices. Insertion/deletion errors, on the other hand, change the length of the entire sequence and propagate the error to subsequent slices. For the above three types of errors, although the robustness to system errors can be improved by introducing error correction codes such as RS code or low-density parity-check codes, the system robustness cannot be effectively improved when the error rate exceeds the error correction capability of the error correction codes, and the existing error correction codes are not effective in correcting insertion/deletion errors. Therefore, how to improve the robustness of decoding is crucial. In this article, the process of improving the robustness of decoding is divided into two parts, firstly, the Hamming distance is introduced to prevent error propagation, to eliminate most of the errors. Secondly, the errors that cannot be eliminated partially are corrected by RS code.

Figure 5B shows a schematic diagram of the first level of the error correction process, which aims to stop the error propagation. Firstly, the correct DNA sequence is partitioned into 3-base slices, at this time, the Hamming distance between each slice and the coding table code character is 0. Next, a random error is introduced, and after an insertion/deletion operation, the Hamming distance between each slice and the coding table is re-calculated. Unlike Zan *et al.* (2022), our slices do not necessarily mutate in their Hamming distance after introducing the insertion/deletion error, which is because our 3-base coding table has only 32 characters. There are a total of 4^3^ = 64 possibilities for the three bases, so after an error occurs within a slice, there is a certain chance that it will change to a character within the coding table, and if it is within the coding table, then at this point, the slice Hamming distance will still be 0. However, as the error propagates backward, the Hamming distance of the subsequent slices is bound to change. Therefore, in this article, the non-zero Hamming distance is considered the basis for locating the occurrence of the error. The first slice with non-zero Hamming distance is first found and labeled as the first error slice; for insertion errors, the first base within the slice is deleted; for deletion errors, a random base is inserted at the first position of the segment. The purpose of this error correction level is not to recover the slice where the insertion/deletion error occurred but to cut off the effect of the insertion/deletion on subsequent slices.

In summary, the first level of the error correction process is as follows:

Step 1: Split the read sequences into 3-base slices and calculate the Hamming distance between each slice and the code word in the coding table.

Step 2: Firstly, compare the length of this sequence with the standard length of our designed DNA sequence, if it is less than the standard length then assume that deletion error occurs in this sequence, if it is greater than the standard length then assume that insertion error occurs in this sequence. Secondly, find the first non-zero Hamming distance slice and determine it as the first error slice; if an insertion error occurs, the first base of the slice is deleted; if a deletion error occurs, any base is added to the first position of the slice. Finally, the entire sequence is sliced again in units of 3 bases, and the Hamming distance between each slice and the code word of the coding table is recalculated.

Step 3: After step 2, regardless of whether the Hamming distance of the first erroneous slice is restored to 0 or not, this slice is skipped, and the process of step 2 is repeated for the subsequent slices until all slices are traversed.

The RS code added to the DNA sequence was used as the second level of error correction, and to test the effect of the overall error correction scheme, 0.1%–10% mixed errors were randomly introduced into the 2316 base sequences encoded from the Mona Lisa picture with a length of 207 nt. To conform to the error pattern of the Illumina MiSeq platform, the ratio of substitutions to insertions/deletions in the mixed errors was 27:1. As shown in Fig. 6C, when the mixed error rate reaches 2%, more than 90% of the data can still be recovered. Therefore, for the Illumina MiSeq platform, which has the smallest sequencing error rate, our error correction scheme is fully competent.

### 4.3 Comparison with existing schemes

Table 1 lists the encoding potential, net information density, error correction scheme, GC content and maximum homopolymer length of the other six storage schemes compared to the present scheme. The calculation of encoding potential is given by Equation (10).

(10) Dtheory=LbinNDRRColigo×LDRRCinoligo+NBlawatoligo×LBlawatinoligo

**Table 1.** Comparison of the DRRC storage system with the previous storage scheme.

	Input data (Mbytes)	Coding potential (bit/nt)	Net information density (bits/nt)	Error correction/detection	GC content (%) of sequence	Maximum homopolymer length (nt)
Church *et al.*	0.65	1	0.83	NO	2.5–100	3
Goldman *et al.*	0.75	1.58	0.33	Repetition	22.5–82.5	1
Grass *et al.*	0.08	1.78	1.14	RS	12.5–100	3
Blawat *et al.*	22	1.6	0.92	BCH + RS + CRC	19–71	3
Erlich and Zielinski	2.75	1.98	1.57	Fountain	40–60	4
Chen *et al.*	0.318	1.95	1.89	RS	40–60	4
This work	0.836	1.66	1.352	Hamming distance + RS	50	3

$L_{\mathrm{bin}}$ represents the length of the original binary sequence, $N_{\mathrm{DRR}C_{\mathrm{oligo}}}$ and $N_{\mathrm{Blawa}t_{\mathrm{oligo}}}$ denote the number of oligonucleotide sequences encoded by the present scheme and the Blatwat scheme, respectively, and $L_{\mathrm{DRR}C_{\mathrm{inoligo}}}$ and $L_{\mathrm{Blawa}t_{\mathrm{inoligo}}}$ denote the lengths of information-carrying portions of oligonucleotide sequences encoded by the present scheme and the Blatwat scheme, respectively. Taking the example of an input size of 876 960 bytes of original data, using the above formulas, our scheme still has a coding potential of 1.66 bit/nt. The calculation of net information density should also consider the index part and error correction code part in the oligonucleotide sequences. Our scheme’s final net information density is 1.352 bit/nt.

DRRC can control DNA encoding constraints fairly precisely compared to other earlier encoding schemes, thereby reducing the complexity of subsequent synthesis and sequencing and avoiding errors to some extent. Some methods use compression of data as part of the encoding process to increase information density, and we emphasize that comparisons should not be made between methods that use compression and those that do not. Moreover, with the rapid growth of information worldwide, it is necessary to store content not only in text form but also in other formats (images, video, audio), which this scheme can do very well.

## 5 Conclusion

In this article, we propose a dual-rule DNA storage system DRRC based on chaotic mapping to control the GC content. A potential advantage of DRRC is that the first position of randomization is introduced to the original sequence before encoding, and the correct and complete original information can only be decoded with the corresponding chaotic sequence parameter, which improves the security of storage. The coding scheme in this article also has a good storage density, which can reach a logical storage density of 1.66 bit/nt without compression mechanism, and supports compression methods such as Huffman, arithmetic coding, etc., which significantly increases the storage capacity.

In terms of robustness, the use of mapping table coding is more or less likely to have some impact on error propagation, and DRRC is no exception. Therefore, to cope with this problem, this article introduces the concept of Hamming distance to detect error fragments and adopts the method of inserting or deleting bases to cut off the error propagation of the error fragments to the subsequent fragments. Simulations have proven that the data recovery rate is effectively improved after adding this mechanism of blocking error propagation. In this article, RS code is also incorporated into the DNA sequence design to improve data recovery. The data recovery rate can be further improved in the future by increasing the sequencing depth with multiple sequence comparisons. We believe this new storage architecture is expected to facilitate the early realization of DNA storage applications.

## Data Availability

The source code for the codec scheme of this paper is available at https://github.com/Mooreniah/DNA-dual-rule-rotary-encoding-storage-system-DRRC.
